# P2X7R Modulates NEK7-NLRP3 Interaction to Exacerbate Experimental Autoimmune Prostatitis via GSDMD-mediated Prostate Epithelial Cell Pyroptosis

**DOI:** 10.7150/ijbs.94704

**Published:** 2024-06-11

**Authors:** Lei Chen, Yi Liu, Shaoyu Yue, Hui Wang, Jia Chen, Wenming Ma, Wenlong Xu, Muyang Xu, Ziqi Chen, Xianguo Chen, Li Zhang, Chaozhao Liang

**Affiliations:** 1 Department of Urology, the First Affiliated Hospital of Anhui Medical University, Hefei, Anhui, China.; 2 Institute of Urology, Anhui Medical University, Hefei, Anhui, China.; 3 Anhui Province Key Laboratory of Urological and Andrological Diseases Research and Medical Transformation, No. 218, Jixi Road, Hefei 230022, Anhui, China.

**Keywords:** Chronic prostatitis, Prostate epithelial cell, Pyroptosis, P2X7R, Disulfiram

## Abstract

Chronic prostatitis is one of the most common urologic diseases that troubles young men, with unclear etiology and ineffective treatment approach. Pyroptosis is a novel model of cell death, and its roles in chronic prostatitis are unknown. In this study, P2X7R, NEK7, and GSDMD-NT expression levels were detected in prostate tissues from benign prostate hyperplasia (BPH) patients and experiment autoimmune prostatitis (EAP) mice. P2X7R agonist, antagonist, NLRP3 inhibitor, and disulfiram were used to explore the roles of the P2X7R-NEK7-NLRP3 axis in prostate epithelial cell pyroptosis and chronic prostatitis development. We found that P2X7R, NEK7, and GSDMD-NT were highly expressed in the prostate epithelial cells of BPH patients with prostatic inflammation and EAP mice. Activation of P2X7R exacerbated prostatic inflammation and increased NLRP3 inflammasome component expressions and T helper 17 (Th17) cell proportion. Moreover, P2X7R-mediated potassium efflux promoted NEK7-NLRP3 interaction, and NLRP3 assembly and activation, which caused GSDMD-NT-mediated prostate epithelial cell pyroptosis to exacerbate EAP development. Disulfiram could effectively improve EAP by inhibiting GSDMD-NT-mediated prostate epithelial cell pyroptosis. In conclusion, the P2X7R-NEK7-NLRP3 axis could promote GSDMD-NT-mediated prostate epithelial cell pyroptosis and chronic prostatitis development, and disulfiram may be an effective drug to treat chronic prostatitis.

## Introduction

As one of the most common urinary tract diseases in young men, chronic prostatitis has a prevalence of 8.4%-25% worldwide 1. Symptoms such as urogenital pain, and sexual and voiding dysfunction trouble most chronic prostatitis patients. Men with long-time chronic prostatitis tend to develop psychiatric problems, including depression and anxiety 2, 3, which severely reduce patients' quality of life. The etiology and pathogenesis of chronic prostatitis remain unclear. Currently, no effective treatment approach has been developed for chronic prostatitis. Thus, deepening understanding of the mechanism underlying chronic prostatitis and exploring targeted drugs for chronic prostatitis is urgent.

Pyroptosis is inflammatory necrotic cell death which is mediated by the gasdermins family. Increasing evidence demonstrates that pyroptosis promotes the development of inflammatory diseases 4, 5. As one of the gasdermin family members, gasdermin D (GSDMD) is regulated by the NOD-like receptor family pyrin domain-containing 3 (NLRP3) inflammasome 6, and upon activation signals, GSDMD is cleaved by caspase-1 into GSDMD-N terminal (GSDMD-NT). GSDMD-NT is inserted into the cell membrane and forms pores, which induce cell pyroptosis 7, 8. Gao *et al.* demonstrated that GSDMD cleavage by NLRP3 could induce acinar cell pyroptosis in acute pancreatitis, and targeting pyroptosis-related pathways provided an approach for acute pancreatitis treatment 9. As the upstream of NLRP3 inflammasome, the purinergic P2X7 receptor (P2X7R) was demonstrated to regulate the NLRP3 inflammasome pathway through potassium (K^+^) efflux 10. P2X7R was an adenosine triphosphate (ATP)-gated ion channel, which can be activated by ATP and BzATP, etc. The P2X7R/NLRP3 pathway has been proven to promote GSDMD-NT-mediated cell pyroptosis to induce neuroinflammation in migraine, and inhibition of P2X7R was warranted for migraine treatment 11. In diabetic retinopathy, Kong *et al.* observed the P2X7R-NLRP3 axis-mediated retinal endothelial cell pyroptosis 12. Our team previously demonstrated that alcohol exacerbated the progression of chronic prostatitis via upregulating the NLRP3 inflammasome 13, and melatonin could alleviate chronic prostatitis development via the SIRT1/NLRP3 pathway 14. Although the roles of pyroptosis in inflammatory diseases have been clarified a lot, pyroptosis in chronic prostatitis is unclear, and whether P2X7R/NLRP3 could modulate pyroptosis to exacerbate chronic prostatitis development is unknown and deserves further study.

Based on the important roles of pyroptosis in inflammatory diseases, targeting GSDMD-NT-mediated pyroptosis evolved as a novel target for disease treatment. Disulfiram was a commonly used drug to treat chronic alcohol addiction, and Hu *et al.* surprisingly found that disulfiram inhibited GSDMD-NT-mediated pore formation, and administration of disulfiram protected lipopolysaccharide (LPS)-treated sepsis mice 15. In addition, disulfiram inhibited pyroptosis of HK-2 cells to attenuate inflammation and fibrosis in the rat unilateral ureteral obstruction model 16. However, the therapeutic roles of disulfiram in chronic prostatitis remain unknown, which may be another approach for chronic prostatitis management.

Non-obese diabetic (NOD) mice have been proven to be more genetically susceptible to autoimmune diseases, including ooforitis, type 1 diabetes, sialilitis, thyroiditis, adrenalitis, and prostatitis 17-19. NOD mice are widely applied to establish the experimental autoimmune prostatitis (EAP) model and explore the pathogenesis of chronic prostatitis 13, 20, and compared to BALB/C and C57BL/6 mice, more dramatic immune responses and severe prostate histopathological manifestations are observed in NOD mice, which possessed almost all characteristics of chronic prostatitis, from prostate inflammation to pelvic pain symptoms, and increased cytokines levels 18, 20, 21.

T helper 17 (Th17) cells, a subpopulation of CD4^+^ T cells, can secret interleukin (IL)-17A, IL-17F, and IL-22, and Th17 cell-mediated immune response has been proven to participate in autoimmune disease development 22-24. In chronic prostatitis, Th17 cell-mediated autoimmune response plays an important role in disease development 25. Motrich *et al.* found the Th17 cell-driven immune response in patients with chronic prostatitis 26. Consistently, IL-17A levels and Th17 cell proportion were elevated in EAP mice 20. We have previously demonstrated that Th17 cell proportion was increased in patients with chronic prostatitis 27, and excessively activated Th17 cells promoted chronic prostatitis development 28. IL-1β and IL-18, two members of the IL-1 family cytokines, also participate in the pathogenesis of autoimmune diseases 29, and IL-1β levels were increased in patients with chronic prostatitis 26. In addition, pyroptotic cells could secrete IL-1β and IL-18, and IL-1β was demonstrated to promote Th17 cell differentiation 30-32. Therefore, to elucidate the association among prostate epithelial cell pyroptosis, IL-1β, IL-17A, and IL-18 production, as well as Th17 differentiation, could be helpful in understanding the pathogenesis of chronic prostatitis and identifying therapeutic targets.

Herein, roles of P2X7R-NEK7-NLRP3 axis-mediated prostate epithelial cell pyroptosis in chronic prostatitis were explored, and we found that NLRP3 inflammasome was involved in P2X7R-mediated prostate epithelial cell pyroptosis. Mechanistically, P2X7R-mediated potassium efflux promoted NEK7-NLRP3 interaction to induce NLRP3 inflammasome assembly and activation, which cleaved GSDMD into GSDMD-NT and induced prostate epithelial cell pyroptosis and Th17 cell differentiation. Importantly, we found that disulfiram could ameliorate chronic prostatitis by inhibiting GSDMD-NT-mediated prostate epithelial cell pyroptosis and Th17 cell differentiation, and targeting P2X7R and pyroptosis may be effective in chronic prostatitis treatment.

## Materials and methods

### Clinical samples collection

Under the Declaration of Helsinki Principles, the prostate tissues were collected from benign prostatic hyperplasia (BPH) patients in the First Affiliated Hospital of Anhui Medical University (FAHAMU). BPH patients with urinary retention, incidental prostate cancer, prostate intraepithelial neoplasia after surgery, and urinary tract infection before surgery were excluded from this study. Our study was approved by the ethical committee of FAHAMU (Approval No. PJ 2024-01-65), with written informed consent obtained from these BPH patients. The criterion for assessing the degree of prostatic inflammation in BPH patients was listed in **Table S1** 33.

### Establishment of the EAP model and drug treatment

**Figure 1:**
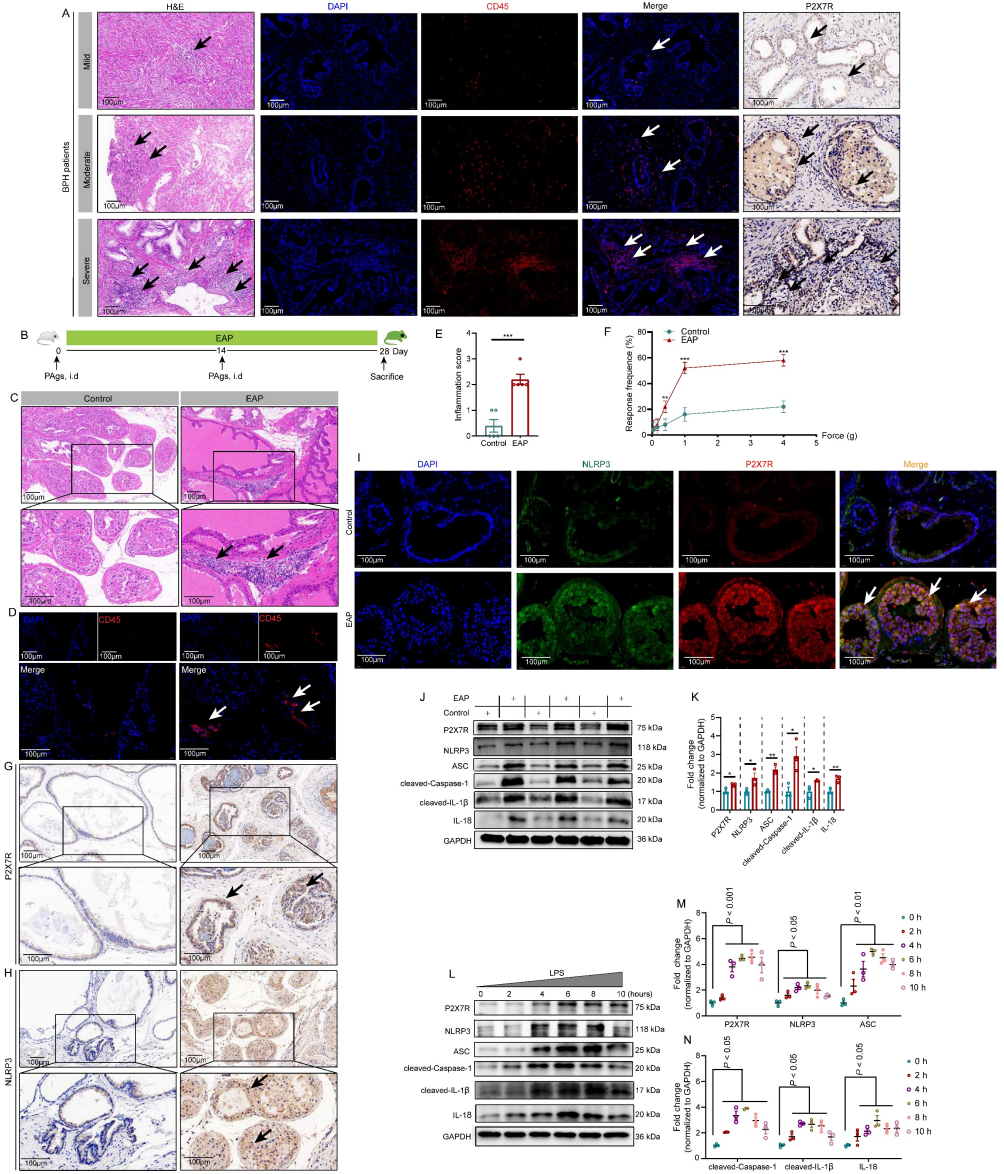
** The highly expressed P2X7R in BPH patients, EAP mice, and LPS-treated RWPE-1 cells.** The results of H&E staining, CD45 immunofluorescence assay, and P2X7R expression by immunohistochemistry in BPH patients with mild, moderate, and severe prostate inflammation (**A**). The processes of EAP model induction through subcutaneous injection of PAgs at day 0 and day 14, and the whole duration was 28 days (**B**). H&E and CD45 staining showed the infiltrated immune cells in control and EAP mice (**C-D**), and the difference in inflammation score and pain response between control and EAP mice (**E-F**). Immunohistochemistry and immunofluorescence showed P2X7R and NLRP3 expression and distribution in the prostate of control and EAP mice (**G-I**), and WB further indicated P2X7R and NLRP3 inflammasome component expressions in the prostate of control and EAP mice (**J-K**) and LPS-treated RWPE-1 cells (**L-N**). *P < 0.05, **P < 0.01, ***P < 0.001, N = 3-5/group. BPH: benign prostate hyperplasia; EAP: experimental autoimmune prostatitis; H&E: hematoxylin and eosin; i.d: intradermally; PAgs: prostate antigens.

The experimental autoimmune prostatitis (EAP) model has been widely used to investigate the pathogenesis and mechanism underlying chronic prostatitis 13, 20, 34. As previously described 13, 20, 34, a total of 50 prostate glands was isolated from Sprague-Dawley rats, and the pooled glands were homogenized in 0.01 M phosphate-buffered saline (PBS, pH 7.2) with protease inhibitors and 0.5% Triton X-100 in the homogenizer (KZ-5F-3D, Servicebio). The homogenate was centrifuged at 10,000 g for 30 min at 4 °C to obtain the supernatant as the prostate antigens (PAgs). PAgs were detected for protein concentration by using the BCA assay kit (P0010S, Beyotime) and stored at -80 °C. We purchased six weeks of NOD mice from the Nanjing University (Nanjing, China). For EAP induction, PAgs or saline solution are mixed with complete Freund's adjuvant (CFA, Sigma-Aldrich). PAgs (300 µg/mouse) emulsified with CFA were intradermally (i.d.) injected in the shoulder (50 µl), the tail base (50 µl), and the right (25 µl) and left foot pad (25 µl) of NOD mice at day 0 and day 14, and NOD mice in the control group were i.d. injected with the same volume of saline solution emulsified with CFA. For drug treatment, BzATP (B6396, Sigma), Brilliant Blue G (BBG, B0770, Sigma), and MCC950 (S7809, Selleck) were dissolved in water, and NOD mice were administrated with BzATP (5 mg/kg, intraperitoneally (i.p.)) 35 or BBG (50mg/kg, i.p.) 11 after the second immunization for consecutive 14 days. MCC950 was administrated (10mg/kg, i.p.) every two days from the second immunization 13. Disulfiram (DSF, HY-B0240, MCE) was dissolved in 10% DMSO and 90% corn oil, which was treated in mice (50mg/kg, i.p.) after the second immunization for a consecutive 10 days, and the control group was treated with 10% DMSO and 90% corn oil 15. The whole process of EAP induction is displayed in **Figure 1B**. This study was approved by the Committee for Animal Care and Use of the Animal Center of Anhui Medical University (Approval No. LLSC20221283).

### Behavioral test

Tactile allodynia and referred hyperalgesia of NOD mice were tested by using von Frey filament to reflect the pelvic pain behaviors of EAP mice 36. As performed in our previous studies 28, 37, at day 28, a force of 0.04, 0.16, 0.4, 1.0, and 4.0 g were tested in the lower abdomen of mice for 10 times, respectively. Response specificity was confirmed based on the following three responses: i) jumping; ii) licking or scratching of the tested area immediately; or iii) retraction of the lower abdomen. The response rate was calculated as times of positive responses / 10.

### Hematoxylin and eosin (H&E) staining

The prostates removed from NOD mice were fixed, dehydrated, and embedded at room temperature. After cutting paraffin-embedded tissues into 4 µm slices, slides were baked at 98 °C for 20 minutes. The slides were then treated with xylene for 10 minutes and alcohol solution (100%, 95%, 75%) for 5 minutes each and washed with distilled water. Afterward, the slides were stained with hematoxylin and eosin, respectively. Images were captured by using a slide scanner (3DHISTECH, Pannoramic MIDI). The degree of prostatic inflammation was identified as previously described, and the criterion 38 for prostatic inflammation identification in EAP mice was listed in **Table S2.**

### RWPE-1 cell culture and reagents

Human prostate epithelial cell (RWPE-1) was purchased from ATCC and cultured in KM medium (Cat# 2101, ScienCell) with 1% of penicillin/streptomycin solution and keratinocyte growth supplement at 4 °C. Lipopolysaccharides (LPS, 2.5ug/ml, L2880, Sigma) was added to the medium to mimic chronic prostatitis *in vitro* 39. RWPE-1 cells were treated with BBG (20 µM) 40, MCC950 (20 µM) 41, or disulfiram (30 µM) 15 two hours before LPS treatment. BzATP (500 µM) was used to treat LPS-primed RWPE-1 cells for 30 minutes 42-44. Detailed information on antibodies and other reagents can be found in **Table S3**.

### Naïve CD4+ T cell isolation and differentiation

According to our previous study 28, the isolated naïve CD4+ T cells were plated in a 24-well plate coated with anti-CD3 and anti-CD28 antibodies. The isolated cells were cultured in 1640 medium. IL-6, IL-23, TGF-β1, anti-IL-4, anti-IFN-γ were added to induce Th17 cell differentiation with or without IL-1β. Cells were harvested at 5 days for flow cytometry.

### Flow cytometry

After incubating with an anti-mouse CD4 antibody for 1 hour at 4 °C, cells were washed with PBS and stimulated with PMA, ionomycin, and monensin for 4 hours at 37 °C. Then, cells were fixed and permeabilized with the Staining-Buffer Set (Cat# 00-5523-00, eBioscience) and incubated with anti-mouse IL-17A antibody for 1 hour at 4 °C, and detected by using a CytoFLEX flow cytometer (Beckman, Americas).

### Immunohistochemistry

The prostate samples sliced were prepared as described in H&E staining. After deparaffinization and rehydration, antigen retrieval was achieved with citrate buffer. Subsequently, 3% H_2_O_2_ was used to destroy endogenous peroxidase. Following blocking non-specific sites by goat serum for 20 minutes, primary antibodies against P2X7R, NLRP3, NEK7, ASC, cleaved-Caspase-1, GSDMD-NT, cleaved-IL-1β, and IL-18 were incubated overnight at 4 °C. Then, sections were incubated with secondary antibodies for 15 minutes, and processed with horseradish peroxidase and 3,3′-diaminobenzidine tetrahydrochloride. Finally, hematoxylin was applied to the counterstain cell nucleus. Images were obtained by a light microscope.

### Immunofluorescence

Slides were prepared as described in immunohistochemistry. After fixation and permeabilization of the samples, slides were incubated with the primary antibodies against P2X7R, NLRP3, ASC, cleaved-Caspase-1, cleaved-IL-1β, GSDMD-NT, and CD45 overnight at 4°C. Subsequently, secondary antibodies were incubated for 2 h. After staining with DAPI, the immunofluorescent signals were captured by using a confocal laser scanning microscopy (LSM 800, ZEISS, German).

### Electron microscopy

A scanning electron microscope (SEM) was used to observe morphology changes in RWPE-1 cells. Pyroptosis of mice prostate epithelial cells was observed under a transmission electron microscope (TEM). For SEM, RWPE-1 cells were seeded on glass slides, after treatment with LPS + BzATP as mentioned above, cells were fixed with 3% glutaraldehyde and 1% osmic acid, respectively. After drying and being coated with platinum, the morphology of RWPE-1 cells was obtained by using the scanning electron microscope (SU8100, Hitachi, Japan). For TEM, prostate tissues (1 × 1 × 1 mm) obtained from EAP and EAP + disulfiram mice were fixed in 3% glutaraldehyde, after washing with PBS, tissues were fixed with 1% osmium tetroxide. Afterward, tissues were cut into 50-60 nm slices for dehydration and insertion. Finally, samples were observed and scanned by using a transmission electron microscope (Hitachi, Japan).

### RWPE-1 cell Hoechst/PI staining

RWPE-1 cells in control, LPS, LPS + BBG, LPS + BzATP, and LPS + BzATP + MCC950 groups were stained with Hoechst 33342/PI reagent. Briefly, cells were stained with Hoechst 33342 and PI for 30 minutes according to the instructions (Solarbio, CA1120) and observed under confocal laser scanning microscopy (LSM 800, ZEISS, German).

### Co-immunoprecipitation (CO-IP)

CO-IP was used to validate the interaction between NEK7 and NLRP3, and all procedures were under the protocol of Immunoprecipitation Kit with Protein A+G Magnetic Beads (P2179S, Beyotime). After washing beads with TBS, the beads were incubated with antibodies against NLRP3, NEK7, or normal rat IgG for 1 hour at RT. The anti-NLRP3/NEK7/IgG binding beads were washed with TBS. The RWPE-1 cells treated with LPS, LPS + BzATP, or LPS + BzATP + KCl were harvested, and cell lysis was incubated with antibodies binding beads at 4 °C overnight. The beads were washed and boiled with loading buffer to perform western blot analysis.

### Western blot

Total proteins were extracted by RIPA lysis solution. After measuring protein concentration with the BCA method, protein samples were boiled with the loading buffer. After running in sodium dodecyl sulfate-polyacrylamide gel, proteins were transferred onto a polyvinylidene difluoride membrane. The membrane was blocked with 5% nonfat milk and incubated with primary antibodies against P2X7R, NLRP3, ASC, cleaved-Caspase-1, cleaved-IL-1β, IL-18, NEK7, and GSDMD-NT overnight. Then, the membranes were incubated with secondary antibodies for 1 h. Finally, the target bands were obtained by using an ECL luminescence reagent using the ChemiScope 5600 chemiluminescence system (Clinx Science Instruments, Shanghai, China). The optical densities (OD) were analyzed by Image J.

### qRT-PCR

Total RNA was extracted by using TRIzol, and cDNA was synthesized using a PrimeScript^TM^ RT reagent Kit (Cat# RR047A, Takara). Real-time PCR was performed with TB Green® Premix Ex Taq™ kit (Cat# RR820A, Takara). Primers used in the study were presented in **Table S4**.

### Intracellular potassium determination

When cells grew to 80% of the 6-cm dishes, BBG was added to the medium to pre-treat RWPE-1 cells for 2 hours for LPS + BzATP + BBG group, and LPS was used to treat cells for 4 hours, and BzATP was added to stimulate cells for 0, 10, 30, and 60 minutes. Then, cells were washed with deionized water. After harvesting cells with nitric acid, intracellular potassium was measured by using an inductively coupled plasma-optical emission spectrum method, and the relative intracellular potassium concentration was calculated.

### ELISA

The concentration of IL-17A (E-EL-M0047c, Elabscience), IL-1β (E-MSEL-M0003, Elabscience), and IL-18 (E-EL-M0730c, Elabscience) were determined in the serum of NOD mice, and the concentration of IL-18 (E-EL-H0253c, Elabscience) and IL-1β (E-EL-H0149c, Elabscience) in the supernatant of RWPE-1 cells with various treatments mentioned above, and IL-17A (E-EL-M0047c, Elabscience) in the supernatant of isolated naïve CD4+ T cells treated as mentioned above were determined.

### Lactate dehydrogenase (LDH) release assay

LDH levels in the medium of RWPE-1 cells were determined by using the LDH Assay Kit. Briefly, cells were seeded on the 96-well plate. LPS and BzATP were used to treat cells for indicated times. After centrifugating for 500g * 4 minutes, 50 µL of the supernatant and 50 µL of work solution were incubated in the plate for 10 minutes at 37 °C. OD 450 nm and OD 600 nm were detected under an Infinite M1000 Pro microplate reader (Tecan).

### Statistical analysis

GraphPad software (Version 8.3) was used to analyze all collected data, and all results were displayed as means ± SEM. Two-tailed Student's *t*-test or one-way ANOVA were used to analyze the data in different groups. *P* < 0.05 was regarded as statistical significance.

## Results

### P2X7R and NLRP3 inflammasome are highly expressed in BPH patients, EAP mice, and LPS-primed RWPE-1 cells

P2X7R expression levels were detected in BHP patient prostates, as shown in **Figure 1A**, compared to BPH patients with mild prostate inflammation, patients with moderate, and severe prostate inflammation exhibited higher expression of P2X7R in the prostate. In EAP mice (**Figure 1B**), infiltrated leucocytes were observed in the prostate (**Figure 1C**), and CD45 staining further demonstrated the inflammatory cell infiltration in EAP mice (**Figure 1D**). EAP mice had a higher inflammation score compared to control mice (**Figure 1E**), and EAP mice had a higher response rate to Von Frey filament than control mice (**Figure 1F**). P2X7R and NLRP3 expression levels were elevated in the EAP group by immunohistochemistry (**Figure 1G-H**), and immunofluorescent assay demonstrated that P2X7R and NLRP3 were co-localized in the prostate epithelial cells of EAP mice, and compared to control mice, EAP mice exhibited higher immunofluorescent density (**Figure 1I**). Moreover, P2X7R and NLRP3 inflammasome component expression levels were elevated in EAP mice by western blot (**Figure 1J-K**). In RWPE-1 cells, P2X7R and NLRP3 inflammasome component expression levels were increased in the presence of LPS stimulation at 0, 2, 4, 6, 8, and 10 hours (**Figure 1L-N**). Hence, the results mentioned above suggested that P2X7R and NLRP3 may play important roles in chronic prostatitis development, which deserves further investigation.

### Activation of NLRP3 inflammasome pathway is involved in P2X7R-mediated EAP development

**Figure 2:**
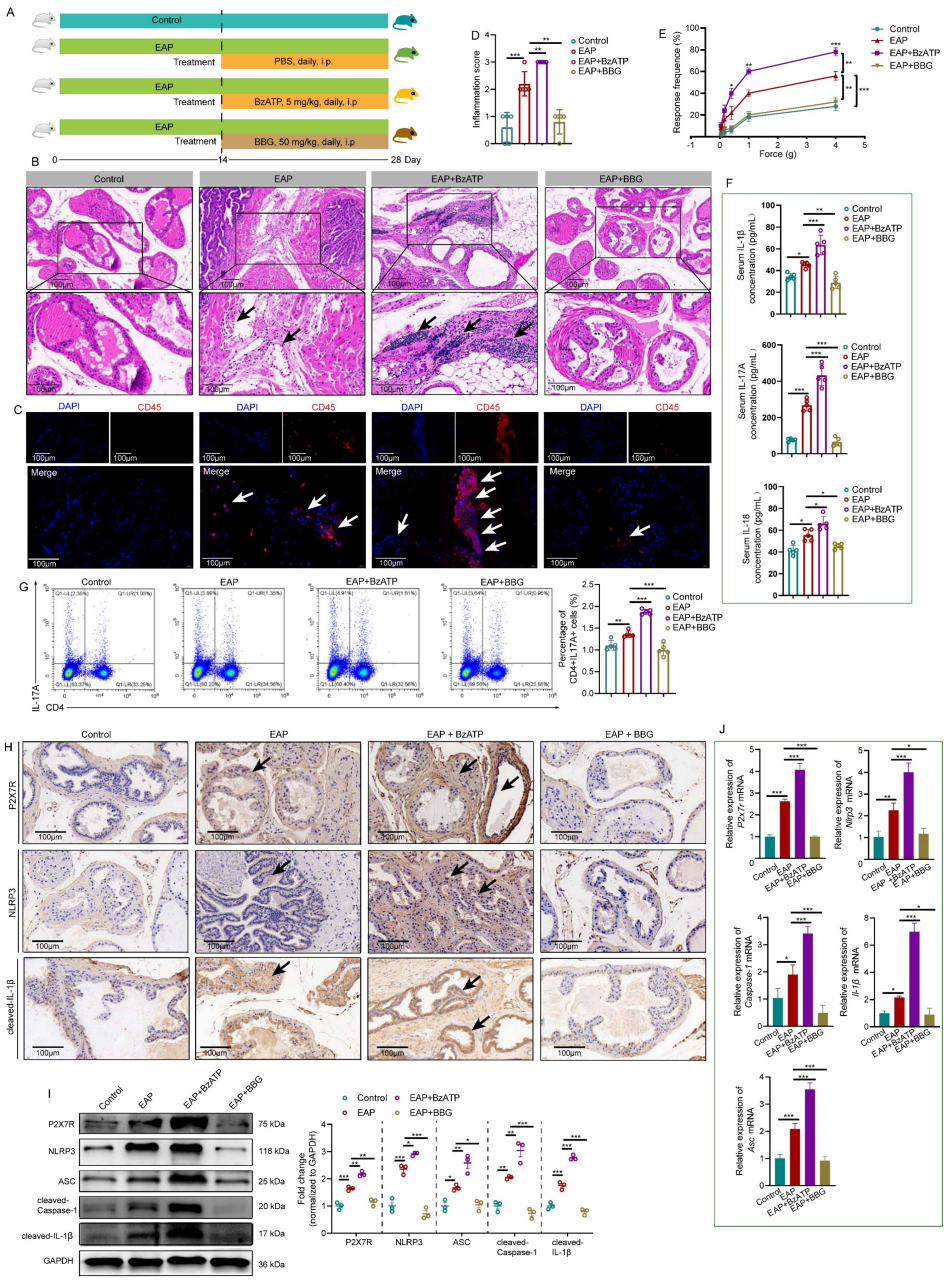
**NLRP3 inflammasome pathway activation is involved in P2X7R-mediated EAP development.** The whole process of BzATP and BBG administration in EAP mice (**A**). The effects of BzATP and BBG on prostate immune cell infiltration (**B**), CD45 immunofluorescence (**C**), inflammation score (**D**), and pain responses (**E**) in EAP mice. Serum levels of IL-1β, IL-17A, and IL-18 in control, EAP, EAP + BzATP, and EAP + BBG mice (**F**). The percentages of Th17 cells in the spleen of control, EAP, EAP + BzATP, and EAP + BBG mice (**G**). Immunohistochemistry and western blot showed P2X7R and NLRP3 inflammasome components expressions in the prostate of mice in control, EAP, EAP + BzATP, and EAP + BBG groups (**H-I**). qRT-PCR showed the expression levels of *P2x7r* and *Nlrp3* inflammasome components mRNA in the prostate of control, EAP, EAP + BzATP, and EAP + BBG mice (**J**). *P < 0.05, **P < 0.01, ***P < 0.001, N = 3-5/group. EAP: experimental autoimmune prostatitis; i.p: intraperitoneally.

Previous studies reported that P2X7R regulated inflammatory responses in various diseases 10, 11, while its role in EAP was unclear. After administrated with BzATP (an agonist of P2X7R) and BBG (an inhibitor of P2X7R) (**Figure 2A**), results showed that activation of P2X7R exaggerated the prostatic inflammation of EAP mice, while inhibition of P2X7R showed the opposite effects (**Figure 2B-C**), which was quantified by the inflammation score (**Figure 2D**). EAP mice treated with BzATP exhibited a higher response rate to Von Frey filament compared to EAP mice alone, and BBG improved EAP mice's responses to Von Frey filament (**Figure 2E**). Compared to control mice, serum IL-1β, IL-17A, and IL-18 levels were elevated in EAP mice, and BzATP treatment further increased the levels of these cytokines, while BBG treatment decreased these cytokine levels (**Figure 2F**). Moreover, compared to control mice, the proportion of spleen Th17 cells increased in EAP mice (**Figure 2G**), and compared to the EAP group, BzATP and BBG treatment increased and decreased Th17 cell proportions, respectively.

Previous studies reported that P2X7R serves as an upstream of NLRP3 10, hence, we speculated that the NLRP3 inflammasome pathway may be involved in P2X7R-mediated EAP development. Results of immunohistochemistry showed that activation of P2X7R by BzATP upregulated P2X7R and NLRP3 inflammasome component expression in EAP mice, while administration of BBG exerted the reverse effects (**Figure 2H, Figure S1A-B**). Western blot analysis also showed that BzATP increased P2X7R and NLRP3 inflammasome component expression levels in EAP mice, while BBG showed the reverse effects (**Figure 2I**). Consistently, *P2x7r*, *Nlrp3*, *Asc*, *Caspase-1*, and *IL-1β* mRNA levels were also increased and decreased after BzATP and BBG administration, respectively (**Figure 2J**). Hence, P2X7R was identified as a new upstream regulator of NLRP3 in chronic prostatitis, and P2X7R-mediated NLRP3 inflammasome activation could induce Th17 cell differentiation and promote chronic prostatitis development.

### Activation of the P2X7R-NLRP3 axis promotes IL-1β and IL-18 secretion in LPS-primed RWPE-1 cells

**Figure 3:**
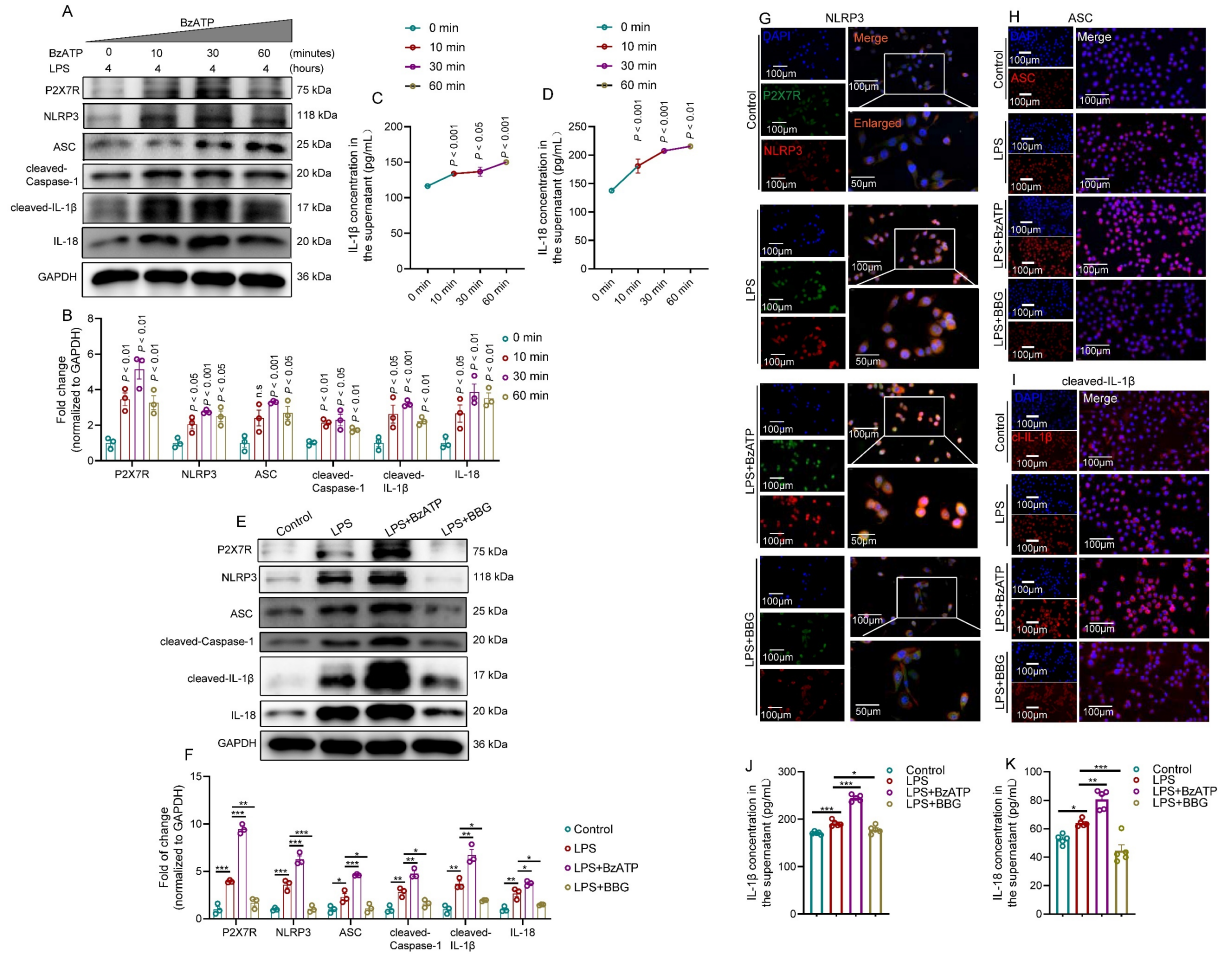
** Activation of the P2X7R-NLRP3 axis promotes IL-1β and IL-18 secretion in LPS-primed RWPE-1 cells.** The effects of BzATP on P2X7R and NLRP3 inflammasome component expressions in LPS-treated RWPE-1 cells at 0, 10, 30, and 60 minutes (**A-B**). The effects of BzATP on supernatant levels of IL-1β and IL-18 at 0, 10, 30, and 60 minutes in the medium of LPS-primed RWPE-1 cells (**C-D**). The effects of BzATP and BBG on P2X7R and NLRP3 inflammasome component expressions in LPS-primed RWPE-1 cells by western blotting assay (**E-F**). Results of the immunofluorescent assay showed the co-localization and immunofluorescent density of P2X7R and NLRP3 inflammasome component in control, LPS, LPS + BzATP, LPS + BBG-treated RWPE-1 cells (**G-I**). ELISA assay showed the supernatant levels of IL-1β and IL-18 in the control, LPS, LPS + BzATP, and LPS + BBG-treated RWPE-1 cells (**J-K**). *P < 0.05, **P < 0.01, ***P < 0.001, N = 3-5/group. cl-IL-1β: cleaved-IL-1β; LPS: lipopolysaccharide.

Because P2X7R and NLRP3 inflammasome were mainly expressed in the prostate epithelial cells of EAP mice, we used LPS-primed human prostate epithelial cells to mimic prostatitis to further validate the effects of P2X7R/NLRP3 pathway on the development of chronic prostatitis. BzATP increased P2X7R and NLRP3 inflammasome component levels in LPS-primed RWPE-1 cells at 0, 10, 30, and 60 minutes (**Figure 3A-B**), and IL-1β and IL-18 levels in the supernatant were increased after BzATP treatment at 0, 10, 30, and 60 minutes (**Figure 3C-D**). Based on the results mentioned above, LPS treatment for 4 hours and BzATP treatment for 30 minutes to RWPE-1 cells were used for the following study. We found that BzATP could upregulate P2X7R and NLRP3 inflammasome component expression levels, and BBG treatment exhibited the reverse effect in LPS-primed RWPE-1 cells (**Figure 3E-F**). Immunofluorescent assay also found that the P2X7R was co-localized with NLRP3 in RWPE-1 cells, and the immunofluorescent densities of P2X7R and NLRP3 inflammasome component were higher in RWPE-1 cells stimulated with LPS+BzATP than LPS treatment alone, and BBG pre-treatment decreased the immunofluorescent density (**Figure 3G-I, Figure S2A-D**). Consistently, LPS treatment increased supernatant levels of IL-1β and IL-18, and BzATP treatment further increased the supernatant levels of IL-1β and IL-18 in LPS-primed RWPE-1 cells, and BBG exerted the reverse effects (**Figure 3J-K**). Hence, the pro-inflammatory roles of the P2X7R-NLRP3 axis were demonstrated in human prostate epithelial cells, which further emphasized the roles of prostate epithelial cells in chronic prostatitis development.

### Blockade of NLRP3 with MCC950 abolishes the pro-inflammatory effects of P2X7R on EAP mice and LPS-primed RWPE-1 cells

**Figure 4:**
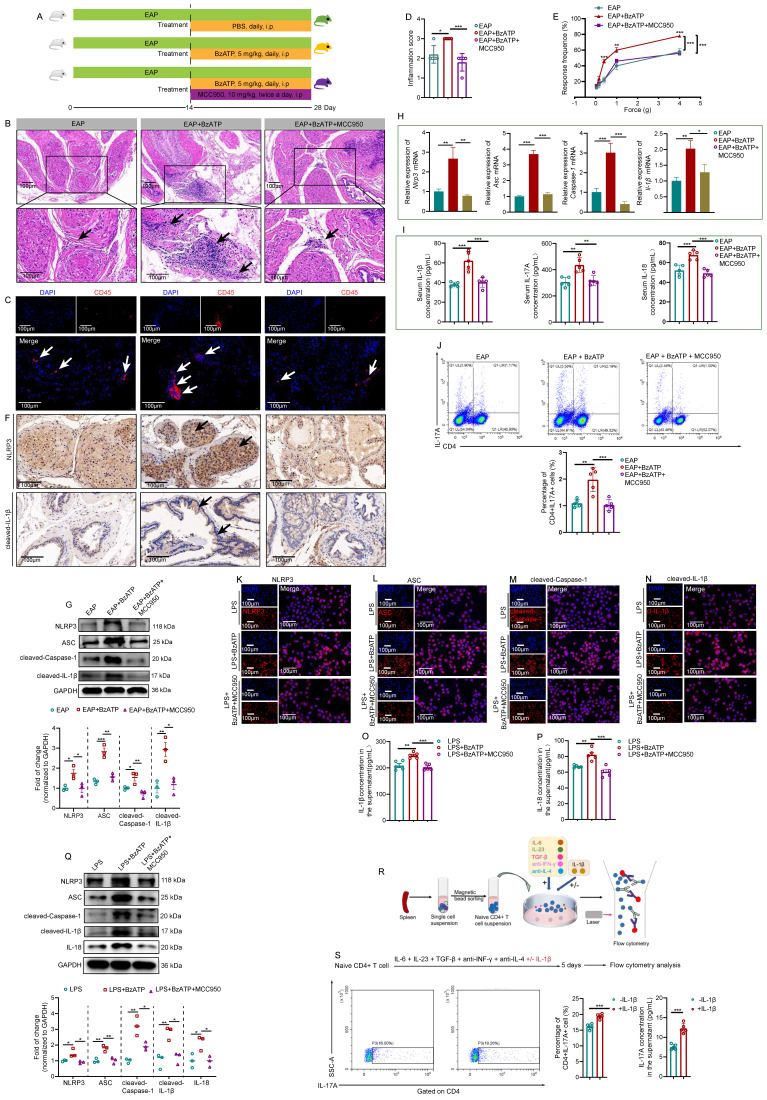
** Blockade of NLRP3 with MCC950 abolishes the pro-inflammatory effects of P2X7R on EAP and LPS-primed RWPE-1 cells.** The whole process of MCC950 administration in EAP mice (**A**). The effects of MCC950 on BzATP-treated EAP mice, including the changes in prostatic inflammation (**B**), CD45 immunofluorescent assay (**C**), inflammation score (**D**), and pain responses to Von Frey filament (**E**). The expression levels of NLRP3, and cleaved-IL-1β in EAP, EAP + BzATP, and EAP + BzATP + MCC950 groups by immunohistochemistry and western blot (**F-G**). qRT-PCR detected the expression levels of *Nlrp3* inflammasome component mRNA in EAP, EAP + BzATP, EAP + BzATP + MCC950 groups (**H**). Serum levels IL-1β, IL-17A, and IL-18 in mice of EAP, EAP+BzATP, and EAP + BzATP + MCC950 groups (**I**). The proportion of spleen Th17 cells in EAP, EAP + BzATP, and EAP + BzATP + MCC950 groups (**J**). The effects of MCC950 on NLRP3 inflammasome component expressions in LPS, LPS + BzATP, LPS + BzATP + MCC950-treated RWPE-1 cells by immunofluorescence (**K-N**). The effects of MCC950 on the supernatant levels of IL-1β and IL-18 in the medium of RWPE-1 cells treated with LPS, LPS + BzATP, and LPS + BzATP + MCC950, respectively (**O-P**). Western blot assay showed NLRP3 inflammasome component expressions in LPS, LPS + BzATP, and LPS + BzATP + MCC950-treated RWPE-1 cells (**Q**). The whole process of isolation of naïve CD4+ T cells (**R**), and the effects of IL-1β on Th17 cell differentiation and IL-17A secretion (**S**). *P < 0.05, **P < 0.01, ***P < 0.001, N = 3-5/group. cl-IL-1β: cleaved-IL-1β; EAP: experimental autoimmune prostatitis; i.p: intraperitoneally.

MCC950, an inhibitor of NLRP3, was applied to investigate the regulatory roles of P2X7R in the NLRP3 inflammasome pathway (**Figure 4A**). Administration of MCC950 attenuated BzATP-exacerbated EAP development, and the prostate inflammation score in NOD mice in the EAP+BzATP+MCC950 group decreased compared to mice in the EAP+BzATP group (**Figure 4B-D**). Consistently, administration of MCC950 decreased the pain response rate of NOD mice in the EAP+BzATP+MCC950 group (**Figure 4E**). Results of immunohistochemistry showed that blockade of NLRP3 with MCC950 attenuated the expression of NLRP3 inflammasome component induced by P2X7R agonist (**Figure 4F, Figure S3A-B**), and western blot analysis also found that elevated expression levels of NLRP3 inflammasome components induced by BzATP were inhibited by MCC950 (**Figure 4G**). The *Nlrp3* inflammasome component mRNA levels were also inhibited by MCC950 (**Figure 4H**). In addition, MCC950 administration decreased serum IL-1β, IL-17A, and IL-18 levels (**Figure 4I**). Besides, compared to the EAP + BzATP group, the percentages of spleen Th17 cells were decreased in the EAP + BzATP + MCC950 group (**Figure 4J**).

The effects of MCC950 on the LPS + BzATP-treated RWPE-1 cells were also investigated. The immunofluorescent assay demonstrated that MCC950 inhibited NLRP3 inflammasome component expressions induced by the P2X7R agonist (**Figure 4K-N**). Moreover, compared to RWPE-1 cells treated with LPS + BzATP, the supernatant levels of IL-1β and IL-18 were also decreased after MCC950 treatment (**Figure 4O-P**). Pre-treatment with MCC950 attenuated the effects of BzATP on NLRP3 inflammasome component expressions in LPS + BzATP-treated RWPE-1 cells by western blot assay **(Figure 4Q)**. In addition, we also investigated the roles of IL-1β in Th17 cell differentiation. Our results showed that IL-1β promoted Th17 cell differentiation *in vitro* (**Figure 4R-S**). Hence, upregulation of P2X7R activated NLRP3 inflammasome pathway in EAP mice and LPS-primed RWPE-1 cells, and blockade of NLRP3 abolished the effects, which demonstrated that P2X7R could promote the development of EAP, and the NLRP3 inflammasome pathway was involved in this process, and P2X7R may be a potential target in EAP treatment.

### P2X7R-mediated K^+^ efflux enhanced NEK7-NLRP3 interaction and exacerbated EAP development

**Figure 5:**
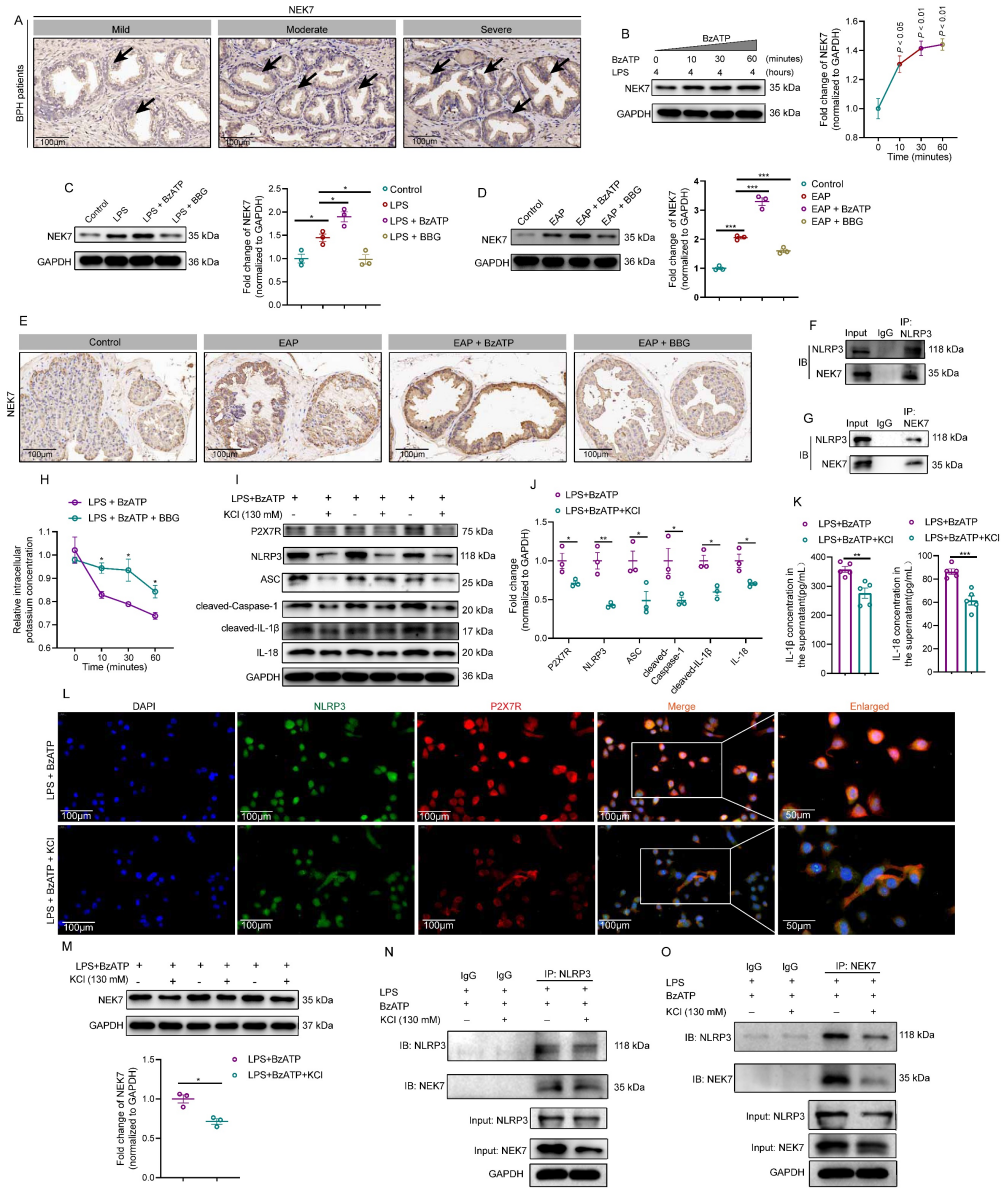
** P2X7R-mediated K^+^ efflux enhanced NEK7-NLRP3 interaction and exacerbated prostate inflammation.** The expression of NEK7 in BPH patients with mild, moderate, and severe prostate inflammation (**A**). The expression levels of NEK7 in the presence of BzATP at 0, 10, 30, and 60 minutes in RWPE-1 cells (**B**). Expression levels of NEK7 in RWPE-1 cells in control, LPS, LPS + BzATP, and LPS + BBG groups (**C**). In EAP mice, the expression levels of NEK7 in control, EAP, EAP + BzATP, and EAP + BBG groups (**D**). Results of immunohistochemistry showed the expression of NEK 7 in control, EAP, EAP + BzATP, and EAP + BBG mice (**E**). CO-IP showed the interaction between NLRP3 and NEK7 (**F-G**). Changes in intracellular potassium concentration between LPS + BzATP and LPS + BzATP + BBG-treated RWPE-1 cells (**H**). Elevation of extracellular potassium concentration influenced the expression levels of P2X7R, NLRP3, ASC, cleaved-Caspase-1, cleaved-IL-1β, and IL-18 in LPS + BzATP-treated RWPE-1 cells (**I-J**). The effects of elevated extracellular potassium concentration on IL-1β and IL-18 secretion from LPS + BzATP-treated RWPE-1 cells (**K**). P2X7R and NLRP3 immunofluorescence density in LPS + BzATP and LPS + BzATP + KCl groups (**L**). Elevated extracellular potassium concentration affected the expression of NEK7 in LPS + BzATP-treated RWPE-1 cells (**M**). Results of CO-IP showed the effects of high extracellular potassium on the interaction between NEK7 and NLRP3 in LPS + BzATP-treated RWPE-1 cells (**N-O**). *P < 0.05, **P < 0.01, ***P < 0.001, N = 3-5/group. BPH: benign prostate hyperplasia; EAP: experimental autoimmune prostatitis; KCl: potassium chloride; LPS: lipopolysaccharide.

Previous studies found that P2X7R served as an ion channel, and ATP could activate P2X7R and induce potassium efflux 45. He *et al.* reported that NEK7 was a mediator of K^+^ efflux-mediated NLRP3 activation 46. However, the effects of P2X7R-mediated K^+^ efflux on NEK7-NLRP3 interaction were unclear in EAP development, and deepening the relationship among P2X7R activation, K^+^ efflux, and NEK7-NLRP3 interaction in EAP was significant to understand mechanisms underlying EAP development. In BPH patients, patients with moderate and severe prostate inflammation had higher NEK7 expression than patients with mild prostate inflammation (**Figure 5A**). In RWPE-1 cells, BzATP treatment increased NEK7 expression at 0, 10, 30, and 60 minutes, and BBG exerted the reverse effects (**Figure 5B-C**). In EAP mice, western blot and immunohistochemistry analysis further demonstrated that BzATP administration increased NEK7 expression, while BBG showed opposite effects (**Figure 5D-E**). Therefore, NEK7 was involved in P2X7R-mediated EAP development. CO-IP analysis found that NLRP3 could interact with NEK7 in LPS-primed RWPE-1 cells (**Figure 5F-G**). Therefore, NEK7 served as a downstream effector of P2X7R, and P2X7R-mediated NEK7-NLRP3 interaction was involved in EAP development. Subsequently, we found that BzATP decreased intracellular K^+^ concentration in LPS-primed RWPE-1 cells, and the addition of BBG inhibited the decrease in intracellular K^+^ concentration (**Figure 5H**). After increasing the K^+^ concentration to 130mM in the medium, the effects of BzATP on P2X7R and NLRP3 inflammasome component expressions were attenuated (**Figure 5I-J**), indicating that K^+^ efflux participated in P2X7R-mediated NLRP3 inflammasome pathway activation. Moreover, elevated K^+^ concentration in the medium also inhibited IL-1β and IL-18 secretion from prostate epithelial cells (**Figure 5K**). Immunofluorescent assay further demonstrated that NLRP3 and P2X7R expressions decreased after high extracellular potassium treatment in LPS + BzATP-treated RWPE-1 cells (**Figure 5L**). Elevated extracellular K^+^ also decreased NEK7 expression in LPS + BzATP-treated RWPE-1 cells (**Figure 5M**). Results of CO-IP showed that 130mM potassium chloride (KCl) in the medium attenuated the interaction between NEK7 and NLRP3 (**Figure 5N-O**). Hence, activation of P2X7R could induce K^+^ efflux to promote NEK7-NLRP3 interaction, resulting in NLRP3 inflammasome activation to exaggerate EAP development.

### GDSMD acts downstream of the P2X7R-NEK7-NLRP3 pathway to mediate prostate epithelial cell pyroptosis and aggravate EAP

**Figure 6:**
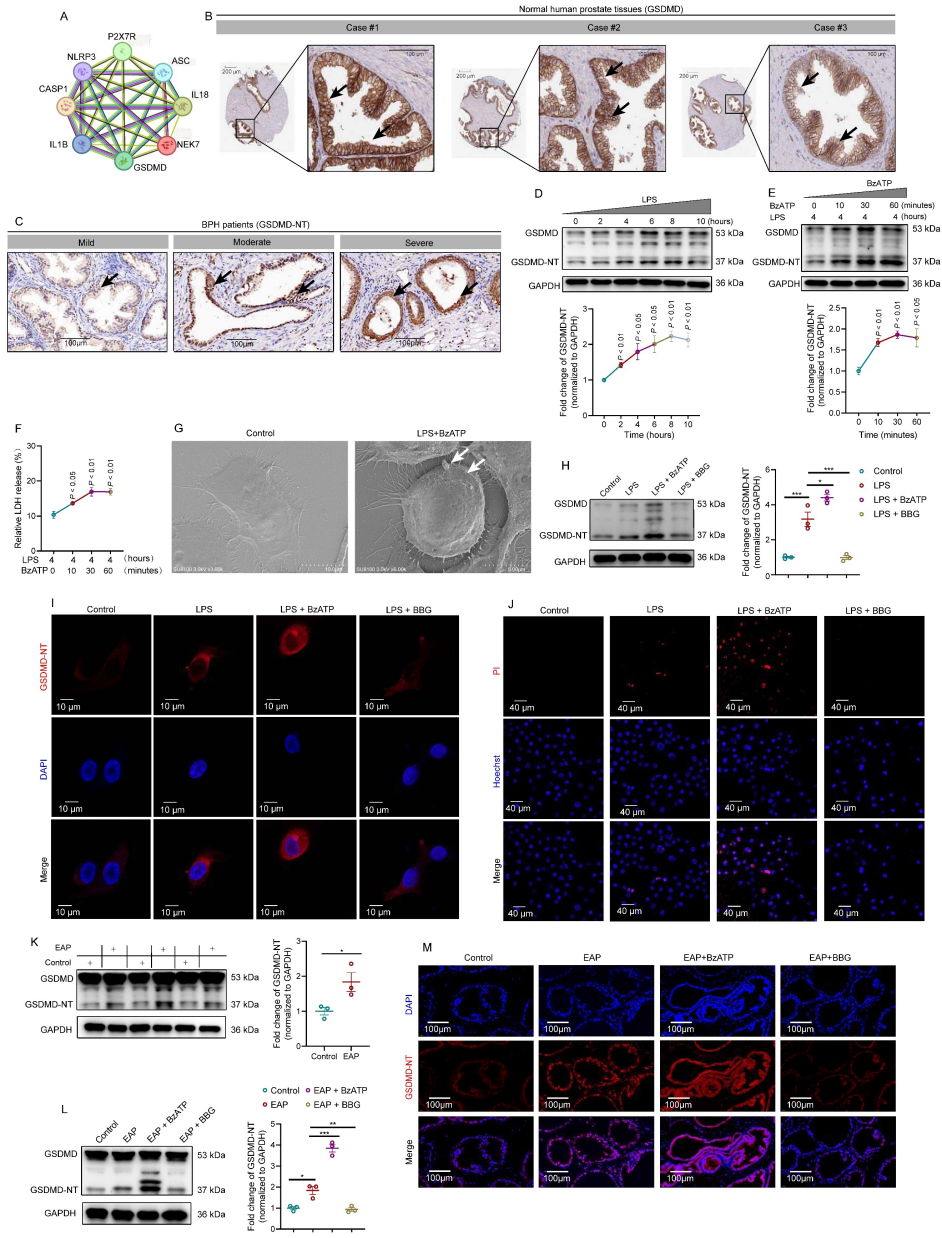
** GDSMD acts downstream of the P2X7R/NLRP3 pathway to mediate prostate epithelial cell pyroptosis and aggravate prostate inflammation *in vivo* and *in vitro.*** Association among P2X7R, NLRP3 inflammasome components, NEK7, and GSDMD in protein-protein interaction analysis (**A**). Expression of GSDMD in normal human prostate by immunohistochemistry in HPA database (**B**). Expression of GSDMD-NT in BPH patients with mild, moderate, and severe prostate inflammation (**C**). The effects of LPS treatment on the expression levels of GSDMD-NT at 0, 2, 4, 6, 8, and 10 hours in RWPE-1 cells (**D**). In LPS-primed RWPE-1 cells, the effects of BzATP treatment on the expression levels of GSDMD-NT at 0, 10, 30, and 60 minutes (**E**). The levels of LDH in the supernatant of RWPE-1 cells treated with LPS + BzATP (**F**). The morphology of RWPE-1 cells in the control and LPS + BzATP groups (**G**). Expression levels of GSDMD-NT in RWPE-1 cells treated with LPS, LPS + BzATP, and LPS + BBG (**H**). Morphology changes and expression levels of GSDMD-NT in RWPE-1 cells treated with LPS, LPS + BzATP, and LPS + BBG (**I**). The proportion of PI-positive RWPE-1 cells in control, LPS, LPS + BzATP, and LPS + BBG groups (**J**). Expression levels of GSDMD-NT in control and EAP mice by western blot (**K**). Expression of GSDMD-NT in control, EAP, EAP + BzATP, and EAP + BBG mice (**L**). Immunofluorescence assay showed the expression of GSDMD-NT in the prostate of control, EAP, EAP + BzATP, and EAP + BBG mice (**M**). *P < 0.05, **P < 0.01, ***P < 0.001, N = 3-5/group. BPH: benign prostate hyperplasia; EAP: experimental autoimmune prostatitis; LPS: lipopolysaccharide.

Generally, activated NLRP3 inflammasome cleaved GSDMD to induce cell pyroptosis 7. Through protein-protein interaction (https://string-db.org/) analysis (**Figure 6A**), we found the association among P2X7R, NLRP3, NEK7, and GSDMD, and we speculated that P2X7R-mediated prostate epithelial cell pyroptosis may promote EAP development. In HPA (https://www.proteinatlas.org/) database, highly expressed GSDMD was detected in normal human prostate tissues (**Figure 6B**). GSDMD-NT was highly expressed in BPH patients with moderate and severe prostate inflammation (**Figure 6C**). Western blot assay indicated that LPS treatment increased GSDMD-NT expression gradually at 0, 2, 4, 6, 8, and 10 hours (**Figure 6D**), and BzATP treatment also led to an increase in GSDMD-NT expression levels gradually at 0, 10, 30, and 60 minutes in LPS-primed RWPE-1 cells (**Figure 6E**). LDH levels in LPS-primed RWPE-1 supernatant were increased gradually after BzATP treatment for 0, 10, 30, and 60 minutes (**Figure 6F**), indicating that LPS + BzATP treatment could decrease cell membrane integrity to permit LDH release. Based on the results mentioned above, LPS treatment for 4 hours and BzATP treatment for 30 minutes were used to induce the pyroptosis of RWPE-1 cells. SEM analysis showed that LPS and BzATP could successfully induce the pyroptosis of RWPE-1 cells, including cell swelling and protrusions in the cell membrane (**Figure 6G**). In RWPE-1 cells, BzATP promoted GSDMD-NT expression, while BBG treatment inhibited GSDMD-NT expression by western blot analysis (**Figure 6H**). Subsequently, we detected the distribution of GSDMD-NT in LPS + BzATP-treated RWPE-1 cells by using confocal laser scanning microscopy to examine GSDMD-NT-induced cell pyroptosis. Results showed that compared to normal cells, LPS + BzATP-treated RWPE-1 cells exhibited pyroptotic changes, and GSDMD-NT was detected to mainly localize in cell membranes (**Figure 6I**), demonstrating that P2X7R-NEK7-NLRP3 axis could promote GSDMD-NT-mediated prostate epithelial cell pyroptosis, leading to EAP development.

Generally, pyroptosis accompanies cell membrane rupture, and Hoechst 33342 and PI could enter the cells. In this study, PI-positive cell proportions increased after LPS and LPS + BzATP treatment, which further demonstrated that LPS and LPS + BzATP could induce the occurrence of RWPE-1 pyroptosis (**Figure 6J**). Besides, GSDMD-NT was overexpressed in EAP mice (**Figure 6K**), and administration of BzATP upregulated the expression of GSDMD-NT in EAP mice, while BBG decreased GSDMD-NT expression levels by western blot analysis and immunofluorescence staining (**Figure 6L-M**).

Moreover, MCC950 attenuated the effects of P2X7R on GSDMD-NT expression in RWPE-1 cells (**Figure S4A-B**), and confocal imaging demonstrated that inhibition of NLRP3 improved the morphology changes induced by LPS + BzATP, which also decreased the expression of GSDMD-NT (**Figure S4C**). MCC950 also decreased PI-positive cell proportion, indicating that cell pyroptosis was inhibited by MCC950 (**Figure S4D**). In addition, in EAP mice, MCC950 also decreased GSDMD-NT expression in mice prostate, and immunofluorescent assay indicated that MCC950 inhibited GSDMD-NT expression in mice prostate epithelial cells (**Figure S5A-C**). Hence, GSDMD-NT serves as the effector of the P2X7R-NEK7-NLRP3 pathway to permit IL-1β and IL-18 secretion from prostate epithelial cells, targeting pyroptosis was warranted for EAP treatment. The aforementioned findings indicated that apart from immune cells, prostate epithelial cells also played a significant role in chronic prostatitis development.

### Disulfiram attenuated prostate inflammation by inhibiting prostate epithelial cell pyroptosis *in vivo* and *in vitro*

**Figure 7:**
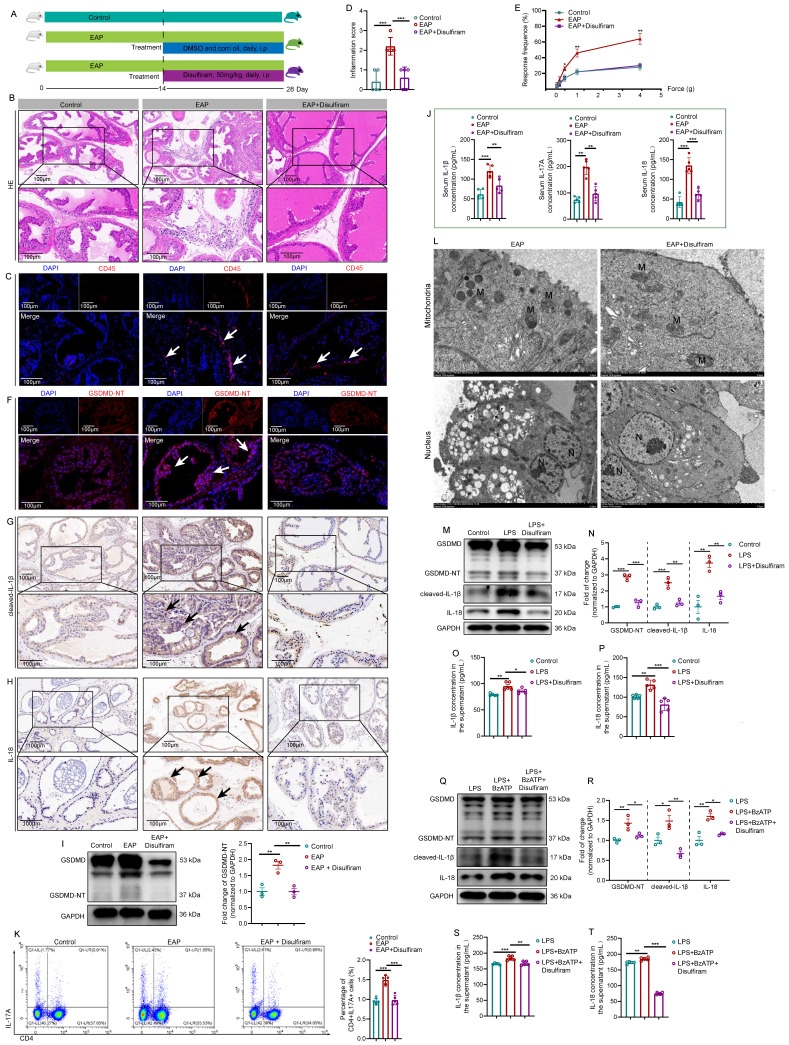
** Disulfiram ameliorates prostate inflammation by inhibiting prostate epithelial cell pyroptosis *in vivo* and *in vitro*.** The Whole process of disulfiram administration in EAP mice (**A**). The effects of disulfiram treatment on prostate inflammation, inflammation score, and pain responses in EAP mice (**B-E**). The expression of GSDMD-NT (**F and I**), cleaved-IL-1β (**G**), and IL-18 (**H**) in mice prostate of control, EAP, and EAP + disulfiram groups. The effects of disulfiram on the serum levels of IL-1β and IL-18 in the control, EAP, EAP + disulfiram groups (**J**). Spleen Th17 cell proportion changes after disulfiram treatment (**K**). The morphological changes in mouse prostate epithelial cells after disulfiram treatment by TEM (**L**). The effects of disulfiram on the expression levels of GSDMD-NT, cleaved-IL-1β, and IL-18 in RWPE-1 cells in control, LPS, and LPS + disulfiram groups (**M and N**), and the changes in the supernatant levels of IL-1β and IL-18 after disulfiram treatment in RWPE-1 cells (**O and P**). Changes in the expression of GSDMD-NT, cleaved-IL-1β, and IL-18 in the LPS, LPS + BzATP, and LPS + BzATP + disulfiram groups in RWPE-1 cells (**Q and R**), and the supernatant levels of IL1β and IL-18 in these three groups (**S and T**). *P < 0.05, **P < 0.01, ***P < 0.001, N = 3-5/group. EAP: experimental autoimmune prostatitis; i.p: intraperitoneally; LPS: lipopolysaccharide; M: mitochondria; N: nucleus.

Currently, inhibitors of pyroptosis including disulfiram 15 and necrosulfonamide 47 have been demonstrated to alleviate inflammatory cell death and sepsis by binding to GSDMD directly to disrupt cell pyroptosis. We explored the therapeutic effects of disulfiram on EAP (**Figure 7A**). In EAP mice, administration of disulfiram attenuated the prostatic inflammation (**Figure 7B-C**), and inflammation score and responses to Von Frey filament were also improved (**Figure 7D-E**). GSDMD-NT, IL-1β, and IL-18 expressions decreased in the presence of disulfiram treatment (**Figure 7F-H**).

**Figure 8:**
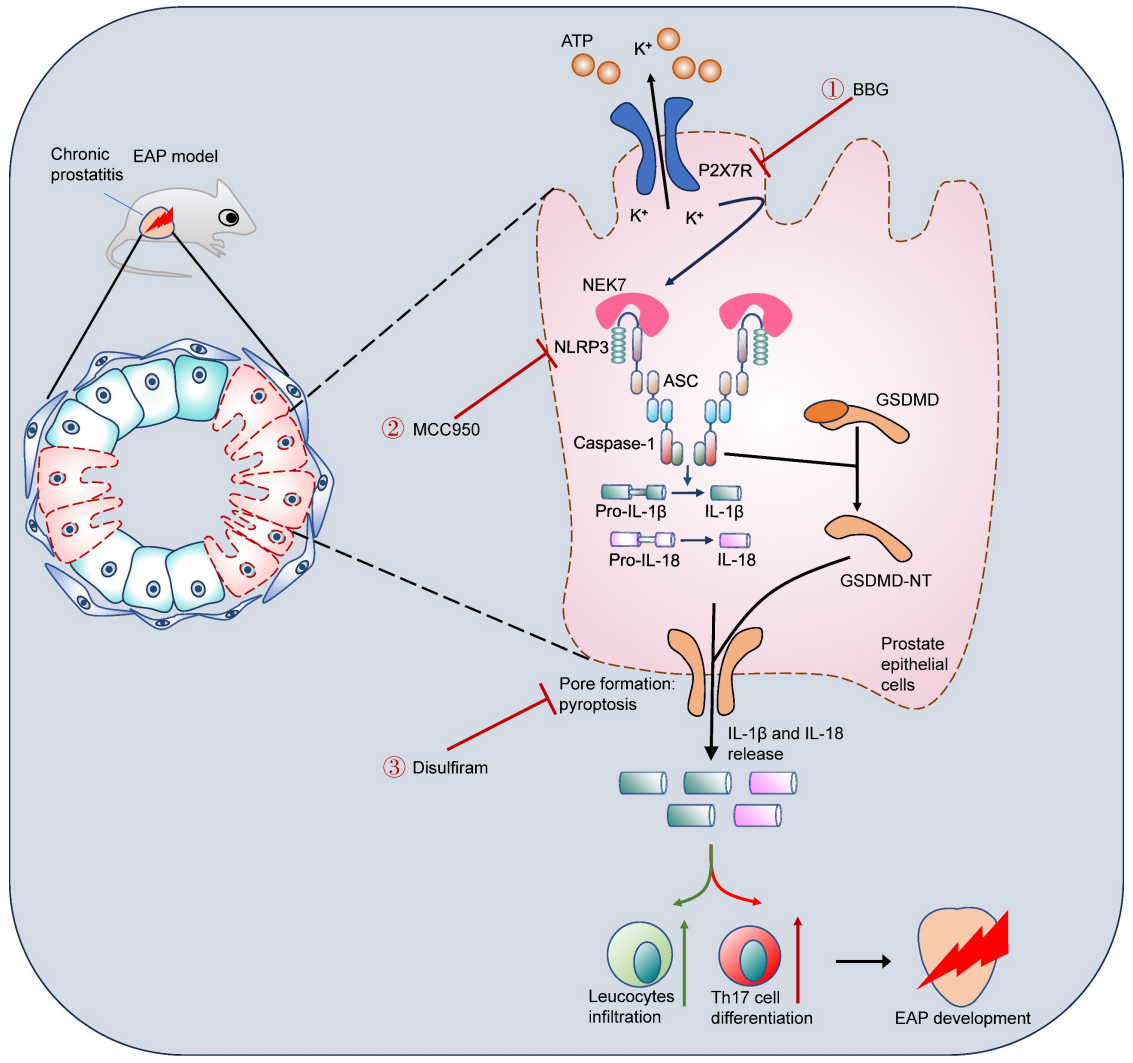
** Schematic illustration of P2X7R-mediated prostate epithelial cell pyroptosis in EAP development.** Upregulation of P2X7R promoted NEK7-NLRP3 interaction and NLRP3 inflammasome assembly and activation, which cleaved GSDMD into GSDMD-NT, and induced cell membrane pore formation and prostate epithelial cell pyroptosis. Targeting P2X7R (BBG), NLRP3 (MCC950), and GSDMD (disulfiram) could alleviate prostate epithelial pyroptosis and elicit important roles in chronic prostatitis treatment. EAP: experimental autoimmune prostatitis; GSDMD: gasdermin D; GSDMD-NT: GSDMD-N terminal.

Western blot further demonstrated that disulfiram could decrease the expression of GSDMD-NT (**Figure 7I**). In addition, serum IL-1β, IL-17A, and IL-18 levels were also decreased in response to disulfiram treatment (**Figure 7J**). Flow cytometry analysis showed that administration of disulfiram decreased spleen Th17 cell proportion (**Figure 7K**). TEM showed that the mitochondria were swelled and dilated, and the chromatin became condensed in prostate epithelial cells of EAP mice, and disulfiram attenuated these pyroptosis-related morphology changes (**Figure 7L**). In RWPE-1 cells, disulfiram downregulated GSDMD-NT expression levels in LPS-primed cells (**Figure 7M-N**), and disulfiram also decreased the supernatant IL-1β and IL-18 levels (**Figure 7O-P**). Moreover, disulfiram also decreased the expression of GSDMD-NT in LPS + BzATP treated RWPE-1 cells (**Figure 7Q-R**), and IL-1β and IL-18 levels in the supernatant were also decreased after disulfiram treatment (**Figure 7S-T**). Hence, P2X7R could promote NEK7-NLRP3 interaction and lead to NLRP3 inflammasome activation, which caused GSDMD-NT-mediated prostate epithelial cell pyroptosis to accelerate EAP development, and disulfiram was effective in inhibiting prostate epithelial cell pyroptosis, which may be a therapeutic approach for EAP treatment (**Figure 8**).

## Discussion

In the present study, P2X7R was demonstrated to promote NEK7-NLRP3 interaction to activate NLRP3 inflammasome and induce prostate epithelial cell pyroptosis, which exacerbated the development of EAP. Our major findings are as follows: (1) P2X7R, an upstream of the NLRP3 inflammasome, promoted EAP development; (2) activation of P2X7R permitted K^+^ efflux, which enhanced NEK7-NLRP3 interaction and induced NLRP3 inflammasome assembly and activation; (3) P2X7R-mediated NLRP3 activation cleaved GDSMD into GSDMD-NT and led to prostate epithelial cell pyroptosis, which permitted IL-1β release and Th17 differentiation; (4) disulfiram attenuated prostate epithelial cell pyroptosis and exhibited potential roles for EAP treatment. Taken together, we found that P2X7R-mediated NLRP3 activation was involved in prostate epithelial cell pyroptosis, and targeting P2X7R and cell pyroptosis was warranted for EAP treatment. Different from previous studies revealing the roles of immune cells in chronic prostatitis development 28, 48, the important roles of prostate epithelial cells in exacerbating chronic prostatitis are elucidated in our study.

Chronic prostatitis brings a heavy burden to patients, with a high incidence rate, and easily relapses 49, and the unclear etiology makes chronic prostatitis difficult to treat, thus, deepening understanding of the mechanisms underlying the development of chronic prostatitis is urgent. We previously found that alcohol exaggerated EAP through the activation of NLRP3 inflammasome, and MCC950 could alleviate alcohol-exacerbated EAP by inhibiting NLRP3 inflammasome 13, and melatonin could effectively alleviate EAP-related pelvic pain and suppress the inflammatory response in EAP via inhibiting the NLRP3 inflammasome 14. Meanwhile, extracorporeal shock wave therapy (ESWT) was reported to improve chronic prostatitis by repressing NLRP3 inflammasome and alleviating apoptosis 50. Hence, NLRP3 inflammasome could exacerbate chronic prostatitis, and targeting NLRP3 inflammasome was warranted for chronic prostatitis treatment. However, the upstream regulator of NLRP3 in chronic prostatitis development is not fully elucidated, and exploring the upstream regulator of NLRP3 may provide novel targets for chronic prostatitis treatment.

In our study, we have noticed that the expression levels of NLRP3 components decreased in the presence of LPS treatment for 10 h, this may be explained by the occurrence of LPS tolerance. LPS tolerance indicates that the cells are hyporesponsive to a relatively long-term LPS treatment with a decrease in cytokines secretion, which widely exists in immune responses in many diseases, including fever and sepsis 51, 52. The mechanisms underlying LPS tolerance are complicated, including changes in specific cell surface receptors, metabolomic changes, mitohormesis reprograms, and transcriptional and epigenomic alterations in tolerant cells 52-55. Hence, combined with the decrease in the expression levels of NLRP3 inflammasome components in the presence of LPS treatment for 10 h, the RWPE-1 cells may be tolerant to LPS treatment.

Pathogen/damage-associated molecular patterns (PAMPs/DAMPs), ATP, etc. were identified as the upstream activator of NLRP3 56. P2X7R was demonstrated to activate NLRP3 inflammasome to enhance collagen-induced arthritis in mice, and sinomenine exhibited therapeutic roles in collagen-induced arthritis by targeting the P2X7R-NLRP3 pathway 57. However, the roles of the P2X7R-NLRP3 axis in chronic prostatitis were unclear. We found that the NLRP3 inflammasome was involved in the P2X7R agonist (BzATP)-exacerbated prostate inflammation in EAP. In RWPE-1 cells, BzATP also promoted NLRP3 inflammasome activation. P2X7R inhibitor (BBG) decreased prostatic inflammation through the inhibition of the P2X7R/NLRP3 axis. MCC950, acting on the NACHT domain of NLRP3, is a small molecule inhibitor of NLRP3 58, and blockade of NLRP3 with MCC950 ameliorated the effects of P2X7R on EAP development. In our previous studies, we found that NLRP3 was involved in alcohol-exacerbated chronic prostatitis 13 and melatonin-attenuated chronic prostatitis 14, while the upstream of NLRP3 was not fully elucidated in chronic prostatitis development. In this study, we identified P2X7R as an upstream of NLRP3, and that suppression of the P2X7R-NLRP3 axis could attenuate EAP development, which may be a therapeutic approach for chronic prostatitis treatment.

As the downstream of potassium efflux, NEK7 is essential for NLRP3 oligomerization and activation 46. Given the significant roles of P2X7R-NEK7-NLRP3 signaling in the development of inflammatory disease, Qishen granule exhibited therapeutic roles in acute myocardial ischemia by targeting P2X7R-NEK7-NLRP3 pathway 59. However, P2X7R-mediated NEK7-NLRP3 assembly and activation were unclear in EAP, and deepening understanding of the underlying mechanisms in EAP was warranted. In EAP mice, we found that NEK7 expression levels increased after BzATP administration, and BBG decreased NEK7 expression in EAP mice. In RWPE-1 cells, BzATP upregulated NEK7 expression after BzATP treatment, and BBG treatment decreased NEK7 expression. We further demonstrated that NEK7 interacted with NLRP3 and activation of P2X7R induced potassium efflux and NLRP3 activation, and elevated extracellular potassium inhibited P2X7R and NLRP3 inflammasome component expressions. Moreover, inhibition of potassium efflux attenuated NEK7-NLRP3 interaction. In neutrophils, potassium efflux was involved in P2X7R-mediated IL-1β production and release to enhance inflammatory responses in diseases 60. Our results demonstrated that P2X7R-mediated potassium efflux and IL-1β production not only occurs in immune cells but also in prostate epithelial cells. Our previous studies elucidated the significant roles of NLRP3 in chronic prostatitis development 13, 14, in this study, we identified how the P2X7R regulated NLRP3 activation to promote chronic prostatitis development, and how potassium efflux played an important role in P2X7R-mediated NLRP3-NEK7 interaction and NLRP3 activation.

GSDMD-NT-mediated cell pyroptosis promotes the development of inflammasome-related diseases 7, while the roles of pyroptosis in EAP were unclear. Our results found that activation of P2X7R increased GSDMD-NT expression, and inhibition of P2X7R had reverse effects *in vivo* and *in vitro*. LPS and BzATP treatment induced the pyroptosis of RWPE-1 cells. Therefore, pyroptosis participated in EAP progression. Pyroptosis inhibitors including disulfiram 15 and necrosulfonamide 47 have been explored to treat inflammatory diseases. Because chronic prostatitis is a common disease with unclear etiology and no effective treatment approach, we used disulfiram to treat EAP and explored effective drugs to alleviate chronic prostatitis. After disulfiram administration, prostatic inflammation and pain response were improved, and GSDMD-NT, cleaved-IL-1β, and IL-18 expressions decreased after disulfiram administration, and disulfiram inhibited IL-1β and IL-18 levels in serum, implying that disulfiram suppressed GSDMD-NT-mediated pore formation in prostate epithelial cells *in vivo* and *in vitro*. Hence, disulfiram may be effective in treating chronic prostatitis, and more studies are needed to further clarify its roles in chronic prostatitis treatment. Previously, immune cells including Th1, Th17, Treg, and macrophage were demonstrated to be involved in chronic prostatitis development 26, 34, 48. As major cellular components in the prostate, although prostate epithelial cells were found to secrete inflammatory mediators in response to *Trichomonas vaginalis* infection 61, 62, the important roles of prostate epithelial cells in chronic prostatitis have been not fully elucidated, and prostate epithelial cell-mediated immune responses may play an important role in chronic prostatitis development. Consistent with our hypothesis, we revealed that the occurrence of prostate epithelial cell pyroptosis promoted chronic prostatitis development. The pyroptotic prostate epithelial cells secreted cytokines including IL-1β and IL-18, and IL-1β enhanced Th17 cell differentiation to exacerbate chronic prostatitis 28.

Deknuydt *et al.* found that IL-1β could induce Treg into Th17 cells by downregulating the transcription factor of Treg cells 30. Because P2X7R enhanced IL-1β secretion via GSDMD-NT-mediated prostate epithelial cell pyroptosis, we proposed that the elevated IL-1β level may increase Th17 cell differentiation to further promote EAP development. We have previously demonstrated that Th17 cell excessive activation exaggerated EAP progression 28. In this study, we found that EAP mice administrated with BzATP increased Th17 cell proportion compared to the EAP group, while BBG exhibited the reverse effects, and *in vitro* assay found that IL-1β enhanced naïve CD4^+^ T cells differentiating into Th17 cells. Hence, our study indicated that P2X7R-mediated IL-1β production and release promoted Th17 cell differentiation and exacerbated EAP development.

## Conclusion

In conclusion, we found that P2X7R-induced potassium efflux could enhance NEK7-NLRP3 interaction, leading to NLRP3 inflammasome activation. P2X7R-NLRP3 axis could induce prostate epithelial cell pyroptosis by converting GSDMD to GSDMD-NT, which formed cell membrane pores and permitted IL-1β and IL-18 production and leakage to promote chronic prostatitis development. Disulfiram could effectively alleviate prostate epithelial cell pyroptosis to suppress the development of chronic prostatitis, which is warranted for chronic prostatitis treatment.

## Supplementary Material

Supplementary figures and tables.

## Abbreviations

ATP: adenosine triphosphate
BPH: benign prostate hyperplasia
CFA: complete Freund's adjuvant
DAMPs: damage-associated molecular patterns
EAP: experiment autoimmune prostatitis
ESWT: extracorporeal shock wave therapy
FAHAMU: First Affiliated Hospital of Anhui Medical University
GSDMD: gasdermin D
GSDMD-NT: GSDMD-N terminal
i.d: intradermally
i.p: intraperitoneally
KCl: potassium chloride
LPS: lipopolysaccharide
NLRP3: NLR family pyrin domain-containing 3
OD: optical densities
P2X7R: P2X7 receptor
PAgs: prostate antigens
PAMPs: Pathogen-associated molecular patterns
NOD: Non-obese diabetic
SEM: Scanning electron microscope
SPF: specific pathogen-free
TEM: Transmission Electron Microscope
Th17: T helper 17
