# Supplementary material for: P2X7R Modulates NEK7-NLRP3 Interaction to Exacerbate Experimental Autoimmune Prostatitis via GSDMD-mediated Prostate Epithelial Cell Pyroptosis
Source: Int J Biol Sci. 2024 Jun 11;20(9):3393–411. doi: 10.7150/ijbs.94704 (PMC11234205; doi:10.7150/ijbs.94704)
Supplement: Supplementary file 1 — Supplementary figures and tables. [file ijbsv20p3393s1.pdf]

P2X7R Modulates NEK7-NLRP3 Interaction to Exacerbate Experimental Autoimmune Prostatitis via GSDMD-mediated Prostate Epithelial Cell Pyroptosis

Lei Chen[#,1,2,3], Yi Liu[#,1,2,3], Shaoyu Yue[#,1,2,3], Hui Wang[1,2,3], Jia Chen[1,2,3], Wenming Ma[1,2,3], Wenlong Xu[1,2,3], Muyang Xu[1,2,3], Ziqi Chen[1,2,3], Xianguo Chen[*,1,2,3], Li Zhang[*,1,2,3], Chaozhao Liang[*,1,2,3]

[1]Department of Urology, the First Affiliated Hospital of Anhui Medical University

[2]Institute of Urology, Anhui Medical University

[3]Anhui Province Key Laboratory of Urological and Andrological Diseases Research and Medical Transformation, No. 218, Jixi Road, Hefei 230022, Anhui, China.

[#] These authors contributed equally to this work.

*Correspondence: Xianguo Chen (cxg7866186@126.com), Li Zhang (lzhang@ahmu.edu.cn), Chaozhao Liang (liang_chaozhao@ahmu.edu.cn)

Tel.: + 86 19955195611, and **Fax.:** + 86 19955195611

Address: No. 218, Jixi Road, Hefei 230022, Anhui, China.

Running title: P2X7R-NEK7-NLRP3 axis promotes EAP development

Word Count: 6,183

Figure number: 8

Supplemental Figure number: 5

Supplemental Table number: 4

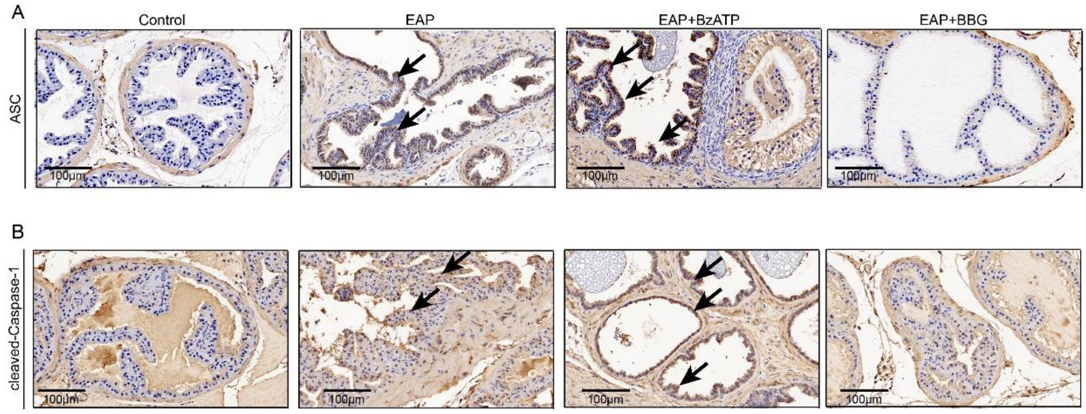

Figure S1. Expression of ASC and cleaved-Caspase-1 in EAP. The expression levels of ASC and cleaved-Caspase-1 in the prostate of control, EAP, EAP + BzATP, and EAP + BBG mice (**A-B**). N = 3/group. EAP: experiment autoimmune prostatitis.

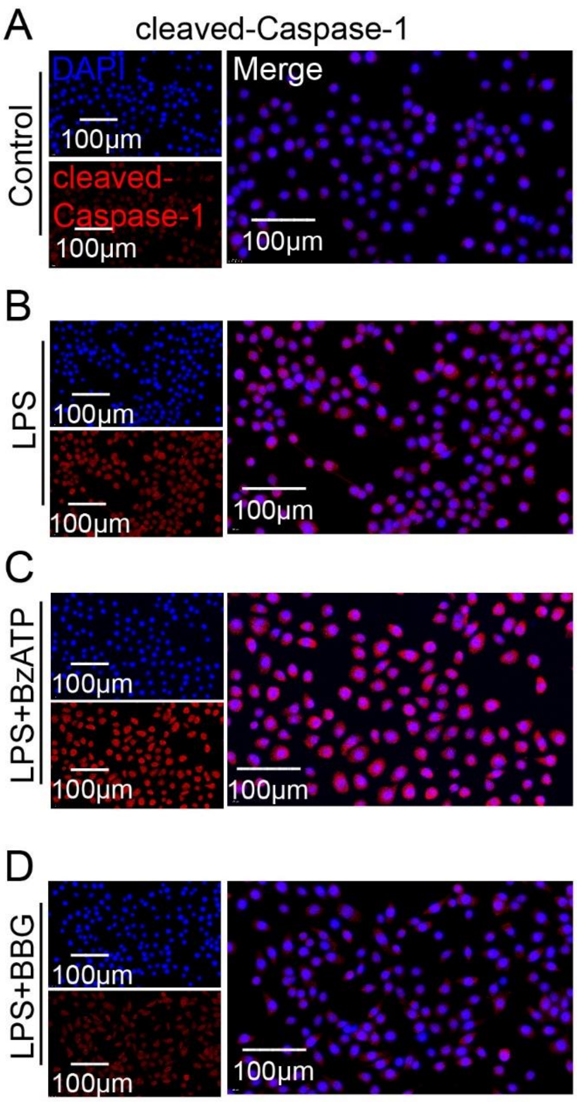

Figure S2. Expression of cleaved-Caspase-1 in RWPE-1 cells. Immunofluorescence density of cleaved-Caspase-1 in RWPE-1 cells in control, LPS, LPS + BzATP, and LPS + BBG groups (**A-D**). N = 3/group. LPS: lipopolysaccharide.

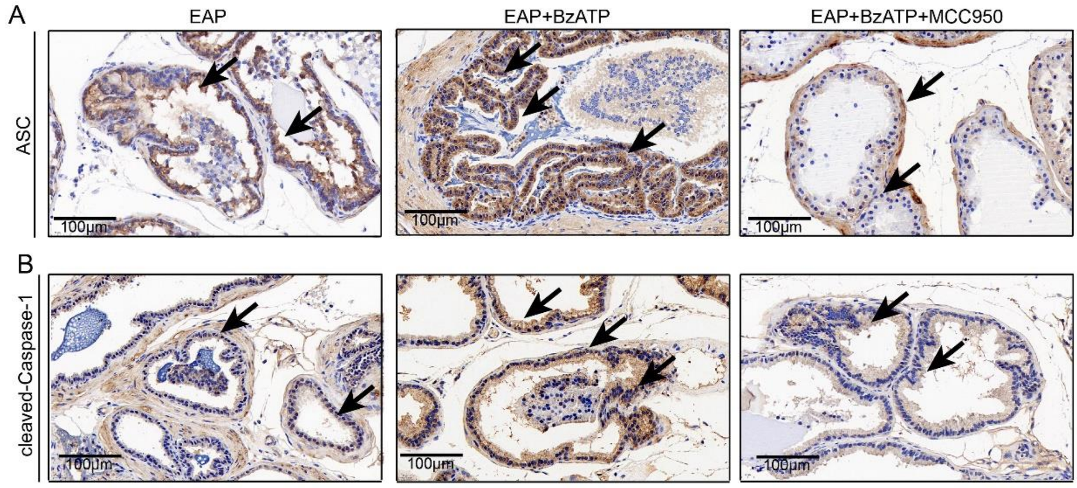

Figure S3. Effects of MCC950 on the expression of ASC and cleaved-Caspase-1 in EAP mice. The expression of ASC and cleaved-Caspase-1 in the prostate of mice in EAP, EAP + BzATP, and EAP + BzATP + MCC950 groups (**A-B**). $^{*}P < 0.05$, $^{**}P < 0.01$, $^{***}P < 0.001$, N = 3/group. EAP: experiment autoimmune prostatitis.

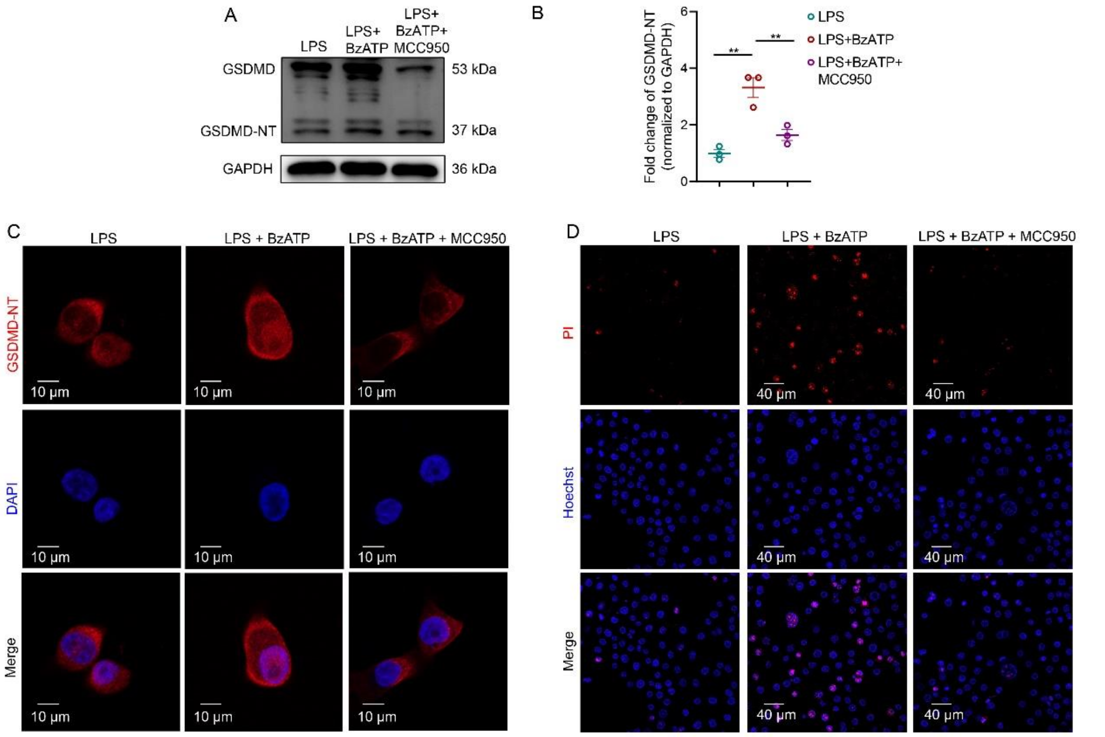

Figure S4. Effects of MCC950 on LPS + BzATP-treated RWPE-1 cells. Expression levels of GSDMD-NT in RWPE-1 cells treated with LPS, LPS + BzATP, and LPS + BzATP + MCC950, respectively (**A-B**). Effects of MCC950 on morphology changes and GSDMD-NT expressions in LPS + BzATP-treated RWPE-1 cells (**C**). Proportion of PI-positive RWPE-1 cells in LPS, LPS + BzATP, and LPS + BzATP + MCC950 groups (**D**). $^{**}P < 0.01$, N = 3/group. GSDMD-NT: GSDMD-N terminal; LPS: lipopolysaccharide.

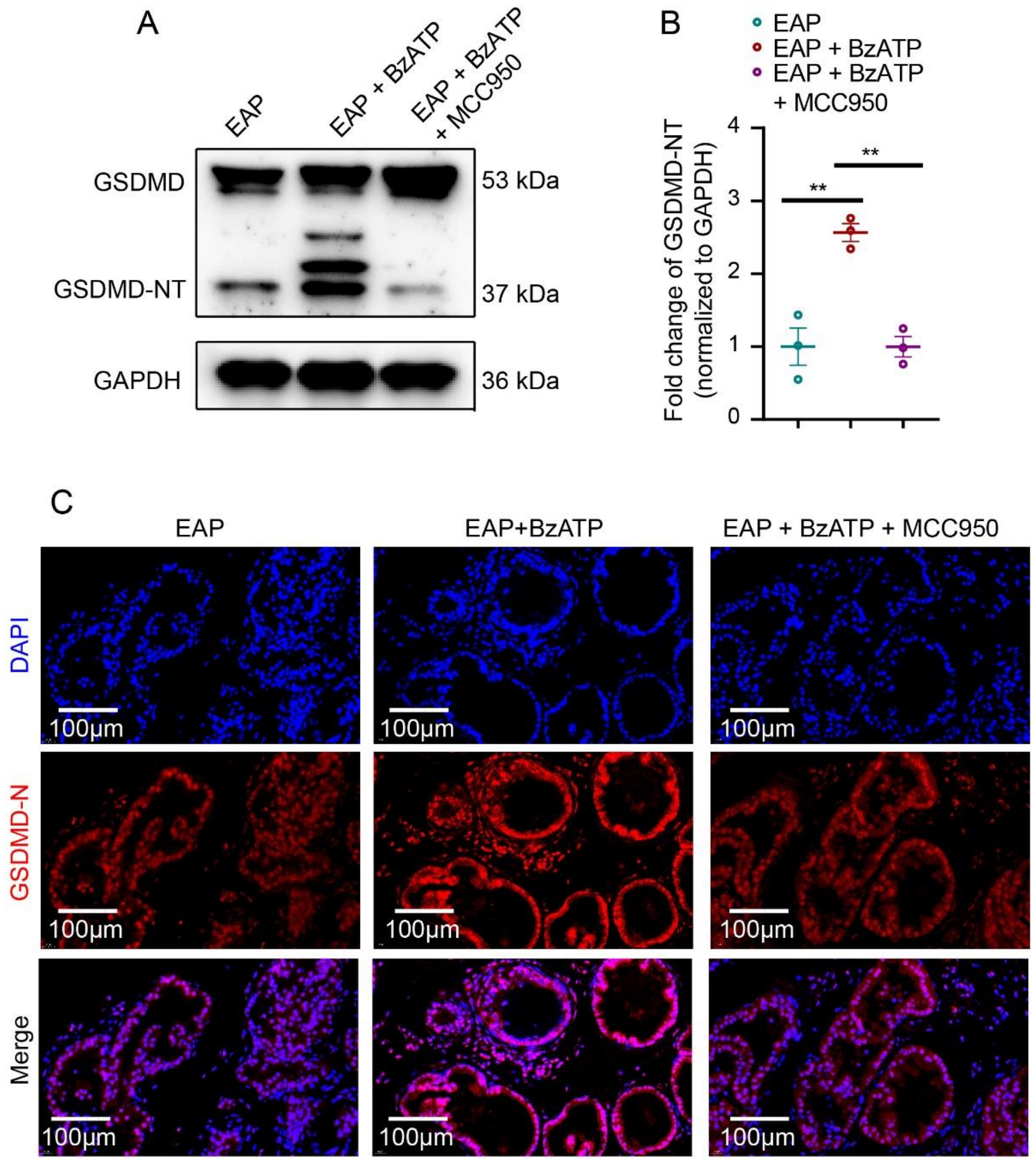

Figure S5. Effects of MCC950 on the pyroptosis of prostate epithelial cells in EAP mice. Expression levels of GSDMD-NT in the prostate of mice in EAP, EAP + BzATP, and EAP + BzATP + MCC950 groups (**A**). Immunofluorescence assay showed the expression of GSDMD-NT in the prostate of mice in EAP, EAP + BzATP, and EAP + BzATP + MCC950 groups (**B**). ***P* < 0.01, N = 3/group. EAP: experiment autoimmune prostatitis.

Table S1. The criterion for identifying prostatic inflammation in BPH patients.

Grade	Morphological description (typical inflammatory cell density, cells/mm2)
1/Mild	Individual inflammatory cells, most of which are separated by distinct intervening spaces (< 100).
2/Moderate	Confluent sheets of inflammatory cells with no tissue destruction or lymphoid nodule/follicle formation (100-500).
3/Severe	Confluent sheets of inflammatory cells with tissue destruction or lymphoid nodule/follicle formation (> 500).

Table S2. The criterion for identifying prostatic inflammation in EAP mice.

Degree (score)	Description
0	no inflammation
1	mild but definite perivascular cuffing with mononuclear cells
2	moderate perivascular cuffing with mononuclear cells
3	marked perivascular cuffing, hemorrhage, and numerous mononuclear cells in the parenchyma

Table S3. Products used in this study.

Products	Cat#	Company
LDH Assay Kit	E-BC-K771-M	Elabscience
Products for naïve CD4+ T cell isolation and flow cytometry		
Naive CD4+ T Cell Isolation Kit	130-104-453	Miltenyi Biotec
LS separation column	130-042-401	Miltenyi Biotec
FITC – Rat Anti-Mouse CD4 antibody	553047	BD bioscience
PE - Rat Anti-Mouse IL-17A antibody	559502	BD bioscience
PMA	CS0001 (50 ng/mL)	Multi Sciences
Ionomycin	CS0002 (500 ng/mL)	Multi Sciences
Monensin	CS0004 (2.5 mg/mL)	Multi Sciences
anti-CD3	BE0001-1 (10 μg/mL)	Bio X Cell
anti-CD28	BE0015-1 (10 μg/mL)	Bio X Cell
IL-6	CG39 (40 ng/mL)	Novoprotein
IL-23	CS31 (40 ng/mL)	Novoprotein
TGF-β1	130-095-066 (1 ng/mL)	Miltenyi Biotec
anti-IL-4	BE0045 (20 μg/mL)	Bio X Cell
anti-IFN-γ	BE0055 (20 μg/ mL)	Bio X Cell

IL-1β	C042 (20 ng/mL)	Novoprotein
Antibodies for western blot, IHC, immunofluorescence, and co-ip		
rabbit anti-CD45 antibody	GB113886	Servicebio
cy3 goat anti-rabbit antibody	GB21303	Servicebio
alexa Fluor 488 goat anti-rabbit antibody	GB25303	Servicebio
rabbit anti-P2X7R antibody	APR-004	Alomone
rabbit anti-P2X7R antibody	28207-1-AP	Proteintec
rabbit anti-ASC antibody	DF6304	Affinity)
rabbit anti-cleaved-Caspase-1 antibody	AF4005	Affinity
rabbit anti-cleaved-IL-1β antibody	AF4006	Affinity
rabbit-anti-IL-18 antibody	DF6252	Affinity
rabbit anti-NEK7 antibody	DF4467	Affinity
rabbit anti-NEK7 antibody	ab133514	Abcam
mouse anti-NEK7 antibody	sc-393539	Santa Cruz
rabbit anti-GSDMD-NT antibody	DF13758 (for mouse)	Affinity
rabbit anti-GSDMD-NT antibody	DF12275 (for human)	Affinity
anti-rabbit secondary antibody	E-AB-1003	Elabscience
anti-mouse secondary antibody	E-AB-1001	Elabscience
rabbit anti-NLRP3 antibody	15101	CST
rabbit anti-NLRP3 antibody	DF7438	Affinity
mouse anti-NLRP3 antibody	BF8029	Affinity
normal rat IgG	2729	CST

Table S4. Primers used in this study.

Genes	Forward	Reverse
P2x7r	GACAAACAAAGTCACCCGGAT	CGCTCACCAAAGCAAAGCTAAT
Nlrp3	ATTACCCGCCCGAGAAAGG	TCGCAGCAAAGATCCACACAG
Asc	CTTGTCAGGGGATGAACTCAAAA	GCCATACGACTCCAGATAGTAGC
Caspase-1	ACAAGGCACGGGACCTATG	TCCCAGTCAGTCCTGGAAATG
Il-1β	GCAACTGTTCCTGAACTCAACT	ATCTTTTGGGGTCCGTCAACT
